# What inhibits working women with mental disorders from returning to their workplace?-A study of systematic re-employment support in a medical institution

**DOI:** 10.1186/s13030-016-0080-6

**Published:** 2016-10-18

**Authors:** Karin Hayashi, Yoichi Taira, Takamitsu Maeda, Yumie Matsuda, Yuki Kato, Kozue Hashi, Nobuo Kuroki, Shuichi Katsuragawa

**Affiliations:** Department of Neuropsychiatry, Sakura Medical Center, Faculty of Medicine, Toho University, Chiba, Japan

**Keywords:** Working women, Psychiatric disorder, Re-work program, Work-family conflict, Work-life balance

## Abstract

**Background:**

It has been customary for working women in Japan to retire when they marry and to devote themselves to household work as well as having children. However, according to a report published by the Ministry of Internal Affairs and Communications in 2013, the number of working women has increased consistently. As more women are advancing into society, they have more options with respect to lifestyle but may encounter new psychological burdens. Therefore, we reviewed trends among participants in a re-work day care program (hereinafter referred to as “re-work program”) to clarify various problems encountered by working women and the prevalence of mental disorders.

**Methods:**

A total of 454 participants (352 males, mean age 46.5 ± 9.4 years; 102 females, mean age 39.8 ± 9.4 years) who participated in our re-work program were included in this study. We reviewed their basic characteristics: life background, clinical diagnoses, outcomes after use of the re-work program, and reasons for failing to return to the workplace or start working where applicable.

**Results:**

The number of female participants was small and accounted for less than one fourth of all participants. As many as 67.3 % of the males succeeded in returning to the workplace, but only 48.0 % of the females were successful. The most common reason for failing to return to the workplace in both sexes was the exacerbation of symptoms; among females, other reasons, such as pregnancy, marriage, and family circumstances, were observed occasionally, but these reasons were not reported by the males.

**Conclusions:**

We found that female-specific problems were not the only issue, but rather work-life balance, relationships in the workplace, and gender differences in work roles could also trigger psychiatric disorders. A deeper understanding of the problems encountered by women in the workforce is important for the treatment of their psychiatric disorders. Therefore, it is considered essential for family members, co-workers, medical staff, and others to understand the various problems encountered by working women. Coping with these problems appropriately will aid in treating mental disorders and creating an environment suitable to prevent their development among women.

## Background

According to the Survey on the State of Employees’ Health by the Ministry of Health, Labour and Welfare, the percentage of Japanese workers who “feel strong uneasiness, worry and stress about their job and working life” reached as much as 60 % in 2007. Moreover, the percentage of “offices where there were workers who had been absent from work for one consecutive month or more or quit their work due to mental health problems reached as much as 7.6 % in the past year”; this percentage increased to 10 % in the 2013 survey [1, 2]. As such, cases of long absence from work or repeated absence from and return to work have become a serious social problem in Japan. To ameliorate such situations, facilities providing day care programs to support return to work (hereinafter referred to as “re-work programs”) have been set up in Japan and now number more than 150.

For many years, it has been customary for Japanese working women to retire when they marry and to devote themselves to household work, as well as bearing and raising children. However, according to a report published by the Ministry of Internal Affairs and Communications in 2013, the number of working women increased consistently from 1987 to 1997. Although the number decreased subsequently for a period, it is presently increasing again. The percentage of working women belonging to the category “married couples with children” tends to increase with age, with 66.2 % matching this description in the age category of 40–44 years. However, more than half of female workers over 35 years of age are non-permanent employees, a percentage that has been increasing over time [3]. Thus, as more women advance within society their lifestyle options increase, but at the same time psychological burdens may be encountered. Prince et al. indicated that women in developed countries are required to do much more than men in various areas of society, including the home [4]. Some reports show that women tend to have less free time than do men [5, 6]. It has also been shown that stress and time restrictions caused by difficulty with work-life balance (harmonization of work and home) can cause mental disorders in women [7]. Indeed, the results of a meta-analysis suggest that impaired work-life balance may cause the development of mental disorders including depression and anxiety disorders [8, 9]. Furthermore, employed women had more job insecurity, lower control, worse contractual working conditions and poorer self-perceived physical and mental health than men did [10].

Therefore the purpose of the present study was to assess the outcomes of a hospital-based re-work day care program for patients who were absent from their work because of a psychiatric disorder. Furthermore, we sought to examine specific factors that obstruct re-work among women. Data from the operation of our re-work program showed a trend such that the percentage of female participants and females returning to work were both low.

## Methods

### Participants

Participants in the re-work program at our hospital took part in a 1-h structured interview with a member of our staff prior to participation. In the interview, we confirmed their psychiatric disorder, clinical condition and explained the re-work program. We also collected information on their life history, work experiences, daily life, and so on. A conference was held based on this information to reach a decision regarding each patient’s participation. In total, 454 participants (352 males, mean age 46.5 ± 9.4 years; 102 females, mean age 39.8 ± 9.4 years) who took part in our re-work program during the period from October 2007 to December 2014 were included in this study. Among them, 58 (35 males, mean age 42.3 ± 8.1 years; 23 females, mean age 37.9 ± 9.0 years) were included who were not trying to return to the workplace or change jobs, but who were trying to start new careers.

### Procedures

We reviewed basic data of the participants, including their life background, psychiatric diagnoses (based on the International Classification of Diseases ICD-10), outcomes after use of the re-work program, and reasons for failing to return to the workplace or starting to work, when applicable.

### Re-work program

**Fig. 1:**
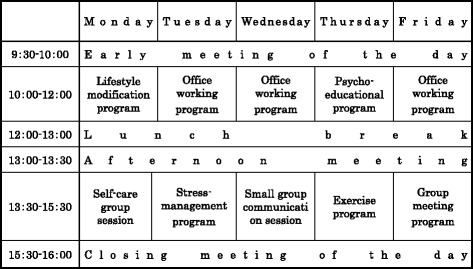
Details of the re-work daycare program. The purposes of our re-work program were (i) to promote a balanced lifestyle for re-work, (ii) to prevent relapse and promote attendance at work, and (iii) to help affected workers assist each other. The program began when the patient’s psychological symptoms had subsided and their motivation to return to work had been recovered. The target period for the process was 3 to 6 months

The purposes of our re-work program were (i) to promote a balanced lifestyle for re-work, (ii) to prevent relapse and promote attendance at work, and (iii) to help affected workers assist each other. The central staff of our program consisted of psychiatric social workers, nurses, and occupational therapists who conducted the program shown in Fig. 1. Clinical psychotherapists and psychiatrists cooperated with these staff members to conduct psycho-education and other programs. The program began when the patient’s psychological symptoms had subsided and their motivation to return to work had been recovered. The target period for the process was 3 to 6 months; however, in some cases could extend to a maximum of 1 year considering the patient’s preparedness for re-work or their workplace situation.

### Data analysis

A chi-squared test was performed with the data divided into men and women. SPSS version 19 was used for analysis, and the significance level was set at *p* < 0.05.

## Results

### Demographic characteristics

As shown in Table 1, less than one fourth of all participants were female (102, 22.5 %), a difference that was significant. Their average age was also significantly lower than that of male participants. Among both males and females, the percentage of participants living alone was less than 20 %, and more than 80 % were living with family. In terms of marital status, there were significantly more females than males who were single or divorced.

**Table 1:** Sex, age, cohabitation status, and marital status of the re-work program participants. Less than one fourth of all participants were female (102, 22.5 %), a difference that was significant. Their average age was also significantly lower than that of male participants. In terms of marital status, there were significantly more females than males who were single or divorced

		Female	Male	All
Number **		102 (22.5 %)	352 (77.5 %)	454
Age	20–29y **	17 (16.7 %)	19 (5.4 %)	36
	30–39y **	36 (35.3 %)	57 (16.2 %)	93
	40–49y	29 (28.4 %)	135 (38.4 %)	164
	50–59y **	19 (18.6 %)	112 (31.8 %)	131
	60y- **	1 (1.0 %)	29 (8.2 %)	30
Living arrangement	alone	15 (14.7 %)	43 (12.2 %)	58
	with family	83 (81.4 %)	307 (87.2 %)	390
	others **	4 (3.9 %)	2 (1.0 %)	6
Marital status	married **	25 (24.5 %)	221 (62.8 %)	246
	single **	65 (63.7 %)	116 (33.0 %)	181
	divorced **	12 (11.8 %)	15 (4.3 %)	27

**p < 0.01

### Clinical diagnoses

Table 2 shows the diagnoses of the mental disorders of the participants. The percentages of participants with F2 (schizophrenia, schizotypal, and delusional) or F3 (affective) disorders were slightly higher among males, but these differences were not significant. The percentage of participants with F4 (neurotic, stress-related, and somatoform) disorders was 22.5 % among females and 19.3 % among males. Thus, adjustment and neuropathic disorders tended to be more frequent among females, whereas somatoform and anxiety disorders tended to be more frequent among males; however, there were no significant differences. Among others disorders, eating disorders were only observed among females whereas organic mental disorders were only observed among males.

**Table 2:** Diagnosis (ICD-10) of re-work program participants. There were no significant difference on diagnoses between men and women

	Female	Male
F2	4 (3.9 %)	17 (4.8 %)
Schizophrenia	3	15
Schizoaffective Disorder	1	2
F3	72 (70.6 %)	261 (74.1 %)
Depressive disorder	59	210
Bipolar disorder	9	45
Dysthymia	0	2
Other affective disorder	4	4
F4	23 (22.5 %)	68 (19.3 %)
Adjustment disorders	8	19
Neurotic disorders	14	36
Somatoform disorders	0	2
Anxiety disorders	1	11
Others	3 (2.9 %)	6 (1.7 %)
Eating disorder *	2	0
Pervasive development disorders	1	2
Organic mental disorders	0	4

*p < 0.05

### Outcomes after completion of the re-work program and reasons for failing to return to the workplace or to start work

Table 3 shows the outcomes after completion of the re-work program. In total, 67.3 % of males succeeded in returning to the workplace in some way; however, only 48.0 % of females were successful, and 37 women (36.3 %) experienced a relapse of their psychiatric symptoms while participating in the program. Both of these differences were significant. In total, 53 female participants (52.0 %) did not return to the workplace or start work by the end of the program.

**Table 3:** Outcome of the re-work program participants. After the re-work program, 67.3 % of males succeeded in returning to the workplace in some way; however, only 48.0 % of females did. Also, 36.3 % of the females experienced a relapse of psychiatric symptoms while participating in the program, but only 20.2 % of males did. Both of these differences were significant

	Female	Male
Succeeded in returning to workplace **	49 (48.0 %)	237 (67.3 %)
Relapse of psychiatric symptoms **	37 (36.3 %)	71 (20.2 %)
Moved to another medical facility	6 (5.9 %)	12 (3.4 %)
Retirement or other	10 (9.8 %)	32 (9.1 %)
Total	102	352

**p < 0.01

Table 4 shows reasons for failing to return to the workplace or start working again among 53 females and 115 males. The most common reason for both sexes was the exacerbation of symptoms. Among females, other reasons such as pregnancy, marriage, and family circumstances were occasionally observed,, while these reasons were not listed for males. On the other hand, among males, some reasons suggested hesitation to return to the workplace, such as “low motivation to return to the workplace” and “problems adapting to the re-work program.”

**Table 4:** The reasons women failed to return to their work place. There were no significant differences between men and women. However, among females, other reasons, such as pregnancy, marriage, and family circumstances, were observed, while these reasons were not listed for males

	Female	Male
Relapse of psychiatric symptoms	37 (69.8 %)	71 (61.7 %)
Moved to another medical facility	6	12
Retired from job	3	5
Some familial problems	2	0
Pregnancy	1	0
To be full-time housewife	1	0
Deviancy	2	2
Job hunting	1	9
Low motivation to return to the workplace	0	6
DC maladaptation	0	3
Waiting to return to the workplace	0	2
Others	0	5
Total	53	115

## Discussion

### Relationship between work and environment

The percentage of female users of the re-work program was significantly smaller compared with male users, and their average age was significantly lower than that of the male participants. This trend can be attributed to the fact that users of the re-work program were predominantly permanent employees, and, as mentioned above, the percentage of non-permanent employees was higher among females and increased with age. Many female users of the re-work program were single or divorced, and a significantly smaller number were married. In Japan, it has been suggested that non-permanent employment, often seen in married women, has increased as a form of “supplemental employment supporting family finance” [11]. Thus, married women have the option of resigning from work if they develop a mental disorder and of devoting themselves to household work, at least temporarily, while single or divorced women do not have the same option and need to return to work to survive financially.

### Mental disorders among working women

With regard to lifetime prevalence rates, it has been shown that the rates of depressive and anxiety disorders are 2 to 3 times higher in women than in men, but there is no sex difference in the rate of schizophrenia. However, among the re-work program users who were participants in this study, as shown in Table 2, there was no significant difference between males and females in terms of clinical diagnoses. Further, with regard to F4 (neurotic, stress-related, somatoform, and other) disorders, there were variations by the respective types of disorder and their prevalence rates were different from the lifetime rates of the general population. One possible reason could be that non-permanent employees and full-time housewives were not included, although many women fall into these categories. Depressive disorders were the most common, among both men and women. It is further noteworthy that eating disorders were observed only among females. In Japan, eating disorders have been observed not only in adolescence but over a wide age range during the past 20 to 30 years. There is a possibility that work-related stress among women may lead to their desire to be thin, which may be related to the development of eating disorders. Uehara et al. reported that the body mass index (BMI) targeted by Japanese women tends to be low, causing concern for future generations [12].

### Reasons for failing to return to the workplaces

As shown in Table 3, among the re-work program participants at our hospital, the percentage of those who succeeded in returning to the workplace was significantly lower among women than among men, and the percentage of those experiencing a relapse of psychiatric symptoms was significantly higher among women.

Close review of possible other reasons showed that “some family problems,” “pregnancy,” and “to be full-time housewife” were listed only for women. No such family-related reasons were listed for the men. Conflict between the family and work domain, which is usually referred to as “work-family conflict,” is considered to lead to stresses in both domains. It has been pointed out that if these stresses become too strong, they may affect mental health adversely [8, 9, 13]. Therefore, the reason why there were more female participants with a relapse of psychiatric symptoms may be related to the presence of their work-family conflict. Thus, medical professionals must take the possibility of these various circumstances into consideration.

## Conclusions

Many problems specific to the population of women in the workforce who develop psychiatric disorders were observed in the current study. However, female-specific problems are not the only issue; work-life balance, relationships in the workplace, and gender differences in work roles can also trigger psychiatric disorders. As for work-life balance, the concept of spillover in addition to the concept of work-family conflict should be considered. It has been reported that the presence of multiple roles may not only have negative effects, such as burdens and conflict, but may also have favorable mutual effects [14].

Based on our results, a deeper understanding of the problems encountered by women in the workforce is important in the treatment of their psychiatric disorders. Therefore, it is considered essential for family members, co-workers, and medical staff, etc., to understand the various problems encountered by working women and to help them appropriately cope with these problems. The ultimate goals should be to create an environment suitable to the prevention of mental disorders and to promote treatment of the mental disorders of women in the workforce.

## Abbreviation

ICD-10: International Classification of Diseases 10
